# Identification of Misfolded Proteins in Body Fluids for the Diagnosis of Prion Diseases

**DOI:** 10.1155/2013/839329

**Published:** 2013-08-21

**Authors:** Francesca Properzi, Maurizio Pocchiari

**Affiliations:** Department of Cell Biology and Neurosciences, Istituto Superiore di Sanità, Viale Regina Elena 299, 00161 Rome, Italy

## Abstract

Transmissible spongiform encephalopathy (TSE) or prion diseases are fatal rare neurodegenerative disorders affecting man and animals and caused by a transmissible infectious agent. TSE diseases are characterized by spongiform brain lesions with neuronal loss and the abnormal deposition in the CNS, and to less extent in other tissues, of an insoluble and protease resistant form of the cellular prion protein (PrP^C^), named PrP^TSE^. In man, TSE diseases affect usually people over 60 years of age with no evident disease-associated risk factors. In some cases, however, TSE diseases are unequivocally linked to infectious episodes related to the use of prion-contaminated medicines, medical devices, or meat products as in the variant Creutzfeldt-Jakob disease (CJD). Clinical signs occur months or years after infection, and during this silent period PrP^TSE^, the only reliable marker of infection, is not easily measurable in blood or other accessible tissues or body fluids causing public health concerns. To overcome the limit of PrP^TSE^ detection, several highly sensitive assays have been developed, but attempts to apply these techniques to blood of infected hosts have been unsuccessful or not yet validated. An update on the latest advances for the detection of misfolded prion protein in body fluids is provided.

## 1. Introduction

There are several forms of Transmissible Spongiform Encephalopathy (TSE) diseases or prion diseases affecting humans and different species of farm and wild animals (i.e., sheep, cattle, and cervids). Some of them have an apparently spontaneous occurrence (i.e., sporadic and genetic TSEs; some forms of atypical bovine spongiform encephalopathy or scrapie), while others are linked to the consumption of prion-contaminated food as in the variant Creutzfeldt-Jakob disease (CJD) [1], feedstuff in bovine spongiform encephalopathy (BSE) [2], or medical and surgical devices in iatrogenic CJD [3]. Transmission of variant CJD via blood transfusion and possibly plasma-derived factor VIII from asymptomatic donors [4] indicates that prion infectivity is present in blood months or years before clinical onset. Thus, the occurrence of epidemics in farm animals and episodes of human cases linked to prion infection pose serious public health issues that are often difficult to solve [5]. An eloquent example is given by the yet unexplained discrepancy between mortality (176 death from 1995 to June 2013) [6] and estimated prevalence data (1 in 4,000 to 1 in 10,000 people) of variant CJD in the British population [7]. This incongruity is causing great concerns because healthy and infected donors who are not promptly identified might transmit disease by blood transfusion, surgical instruments, or plasma-derived products. 

The only validated surrogate marker of infection is the abnormally misfolded isoform of the cellular prion protein (PrP^C^) despite intensive but disappointing search for the identification of other disease-specific biomarkers in easily accessible tissues or body fluids [8, 9]. Misfolded PrP (PrP^TSE^) accumulates in the CNS and other tissues of infected hosts assuming different conformations that are related to the strain of prions [10]. PrP^TSE^ is easily detected by western blot or immunohistochemistry methods after removing the cellular isoform (PrP^C^). Most anti-PrP antibodies, in fact, do not distinguish between PrP^TSE^ and PrP^C^ requiring the removal of the cellular isoform for achieving disease-specific signals. This is usually realised by pretreating samples with proteases (usually proteinase K) that partially digest PrP^TSE^ but completely remove PrP^C^. The use of proteinase K (PK), however, removes fractions of poorly aggregated misfolded PrP^TSE^ that is usually present in blood [11] and likely other body fluids decreasing the chance of detection. Finally, it is still debated whether PrP^TSE^ is unequivocally associated with prion infectivity as there are occasions in which PrP^TSE^ is either not associated with infectivity [12] or absent in infected hosts [13]. Despite these limits, PrP^TSE^ remains the best available choice for confirming the diagnosis of prion diseases and for the identification of prion-associated infectivity in tissues and body fluids. Moreover, the profile that assumes PrP^TSE^ in western blot, reflecting different pathological conformations, is of great help for making a correct molecular diagnosis of sporadic CJD and for differentiating sporadic from variant CJD [14]. 

In the last 15 years several methods have been developed for increasing the sensitivity of PrP^TSE^ detection with the aim of finding a reliable assay for an early diagnosis of prion diseases in easily accessible tissues or body fluids. An overview of these developments is the objective of this work.

## 2. Protein-Misfolding Cyclic Amplification (PMCA)

In 2001, Saborio and colleagues [15] developed a novel protocol for the in vitro amplification of the misfolded prion protein based on the principle that disaggregated PrP^TSE^ incubated in the presence of a large excess of PrP^C^ produces novel PrP^TSE^. Disaggregation of fibrils requires a sonication step, which can be repeated several times, in a cyclic process, to allow sensitive detection of the misfolded PrP of the original seed. The protein-misfolding cyclic amplification (PMCA) was originally developed using hamster brain homogenate and has since been shown to be an efficient method for the amplification of brain PrP^TSE^ of other species including mouse, sheep, cattle, bank voles, cervids, and humans [16–23]. In human samples, the amplification of PrP^TSE^ is strongly influenced by the correct matching of methionine/valine in the 129 residue of PrP, suggesting that this polymorphic site of the protein is important for the amplification of PrP misfolding by the PMCA assay [24–26]. 

Ten cycles of sonication are sufficient to increase the sensitivity of standard western blots from 6–12 picograms to 0.3–0.5 picograms of brain PrP^TSE^  and, with an improved automated protocol which enables a substantial increase in the number of amplification cycles, up to femtogram levels [27]. PMCA is therefore a promising platform for prion diagnosis in body fluids (blood, urine, and CSF) where the level of PrP^TSE^ is estimated in the range of picograms per mL.

The group led by Soto reported the first successful identification of PrP^TSE^ in blood (buffy coat) of scrapie affected hamsters with 89% sensitivity and 100% specificity [27] and positive signals in 50% of samples taken in the preclinical stage of disease as early as 20 days after intraperitoneal 263K scrapie injection [28]. The detection of PrP^TSE^ in blood of preclinical scrapie-infected hamsters is consistent with data on infectivity detection in blood [8]. 

Since then, automated PMCA revealed the presence of PrP^TSE^ in plasma fractions [29], urine [29–31], and cerebral spinal fluid (CSF) [32] of scrapie-diseased hamsters with sensitivity ranging from 50 (plasma) to 100 percent (CSF) (Table 1). In the CSF samples from scrapie-infected hamsters, PMCA was performed by using a further improved protocol (rPrP-PMCA) in which PrP^C^ was replaced by recombinant PrP (recPrP), allowing a sensitivity greater than that observed with previous PMCA protocols [32].

Other than in hamster models, PrP^TSE^ was amplified from blood leukocytes of both naturally [20, 34] and experimentally scrapie-infected sheep [33] where PrP^TSE^ bands were detected as early as 90 days postinoculation and correlated with infectivity titres [33]. On leukocytes of naturally scrapie-infected sheep, PrP^TSE^ was detected in all tested animals with 100% specificity by using an enhanced (i.e., addition of poly-A PMCA) protocol [20]. 

Attempts to detect PrP^TSE^ in blood of other species such as cattle with BSE and cervids with CWD produced negative or controversial results [34, 37, 58]. In patients with various forms of prion diseases, the detection of PrP^TSE^ by PMCA was not attempted (or results were not published) in sample of blood, blood derivatives, plasma, urine, or CSF despite amplification of PrP^TSE^ was successfully reported in human brain samples taken from both sporadic and variant CJDs [16, 24–26]. 

Finally, PMCA amplification of PrP^TSE^ in samples from body fluids, other than blood, taken from prion-infected hosts was successfully achieved in a variety of species and included saliva and urine in sheep with scrapie [36, 36];  saliva, urine, and CSF in cervids with CWD [38, 58]; and CSF and saliva in cattle with BSE [37]. A list of prion-infected body fluids analysed by PMCA with the obtained sensitivity and specificity is shown in Table 1.

In conclusion, PMCA has certainly been a breakthrough for detection of minute amount of PrP^TSE^ that are likely present in body fluids and therefore is a candidate method for developing sensitive tests for the diagnosis of prion diseases in animals and humans. Moreover, the amplified product of PMCA retains the PrP^TSE^ signature of the original seed allowing the molecular diagnosis of CJD in humans and scrapie in sheep with important public health implications. In the last 10 years, PMCA has frequently been modified by addition of poly-A [20] or sulfated dextrans [37], by the use of recombinant PrP instead of brain PrP^C^ [32], or by coupling with sensitive immunoassays [34] that have on one side improved the sensitivity of PrP^TSE^ detection but, on the other hand, made the comparison of data produced by different laboratories difficult. PMCA coamplifies infectivity together with PrP^TSE^ [59, 60] mimicking the disease-specific pathogenic event but requiring safety precautions in diagnostic laboratories. Finally, PrP^TSE^ bands may appear in control preparations after several PMCA cycles [61]. This finding, whether related to *de novo* formation of PrP^TSE^ [20, 60, 61] or cross-contamination of samples [22], raised concern for the reliability of PMCA in diagnostic applications. This inconvenience, however, is easily settled by using low PMCA cycles and appropriate technical tips to avoid possible prion contamination [22].

## 3. Quacking Induced Conversion (QuIC) 

A spin-off of the PMCA method was obtained by substituting sonication with automated tube shaking for the conversion of recPrP substrates [62]. The novel “quacking induced conversion” (QuIC) protocol enables the amplification of 1 femtogram of PrP^TSE^ of scrapie hamster brain homogenate within one day, reducing the complexity and timing of misfolding amplification. Hamster recPrP promotes the conversion of brain misfolded proteins of other species such as sheep with scrapie and humans with sporadic and variant CJDs, regardless of the primary sequence of the PrP^TSE^ seed [41]. Some spontaneous PK-resistant fragments of less than 12 kD are occasionally observed in unseeded control samples [32], but they wane out by reducing the incubation time of the reaction [41].

One of the most significant improvements of misfolding amplification methods was achieved when western blots were replaced by a real-time fluorescent colour reaction (real-time QuIC) [40, 42]. This novel read-out system, based on a fluorescent amyloid-sensitive thioflavin dye (ThT) [63], allowed the implementation of the whole QuIC procedure to a high-throughput 96-well format. The real-time QuIC (RT-QuIC) is an efficient quantitative method for the detection of minute amount of PrP^TSE^ with estimates of the 50% seeding dose (SD_50_) of hamster scrapie brain in the same order of magnitude of infectious doses (LD_50_) [40].

RT-QuIC protocol has been adapted to the detection of brain PrP^TSE^ of other species such as CWD-infected deer, scrapie-infected sheep, and sporadic CJD patients by using species-specific recPrP [40, 42, 64]. Full-length human recPrP and both truncated and full-length hamster recPrPs are efficient substrates for the amplification of PrP^TSE^ in sporadic CJD brain irrespective of the 129 codon phenotypes [43, 64]. A note of disappointment is that the efficacy of variant CJD brain in seeding RT-QuIC reaction is consistently lower than sporadic CJD samples [64].

**Figure 1:**
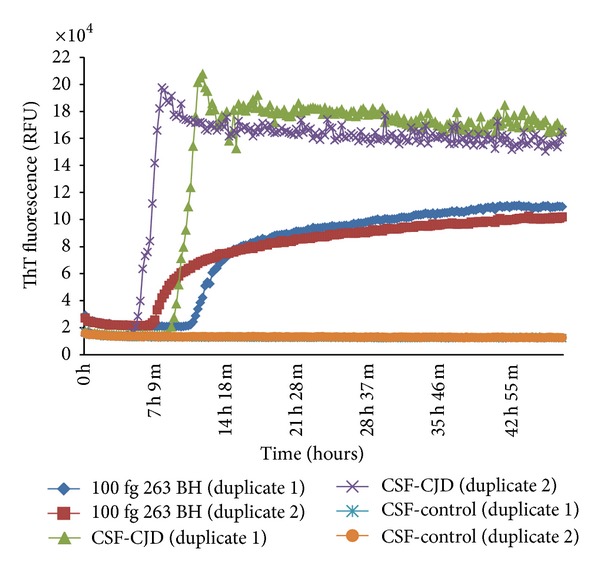
RT-QuIC reactions seeded with 15 *µ*L of human CSF samples from one Italian sporadic CJD patient (12076) and one non-CJD control (13004). 100 fg of 263K prion-infected hamster brain homogenate were used to seed positive control reactions. Each sample was processed in duplicate.

The presence of PrP^TSE^ in the CSF by the QuIC assay was initially revealed by Atarashi and colleagues [62] in 263K-scrapie infected hamsters and Orrú and colleagues [41] in scrapie-infected sheep. In 2010, Wilham and colleagues [40] revealed the presence of PrP^TSE^ in the CSF of 263K scrapie-infected hamsters by RT-QuIC and estimated a titre of about 10^−2^ SD_50_ per *µ*L. CSF samples from control animals did not revealed any presence of PrP^TSE^ indicating a high specificity of the assay. These encouraging results on the CSF of scrapie-infected host promoted further studies in patients with various forms of prion diseases. A blinded experiment was initially performed on 30 CSF samples of definite sporadic CJD patients provided by the Australian National CJD Registry and 155 controls (25 suspected CJD cases and 130 neurological controls) achieving 87.5% specificity and 100% sensitivity. CJD cases were positive irrespectively of 129 codon genotypes [42]. Similarly, McGuire and colleagues [43] screened CSF samples from sporadic CJD patients provided by the National Creutzfeldt-Jakob Disease Research & Surveillance Unit, Edinburgh, UK, including all three 129-codon genotype and obtaining 99% specificity and 94% sensitivity. In the same study, the specificity and sensitivity of the 14-3-3 protein, a surrogate marker currently used for the diagnosis of sporadic CJD, were 65% and 94%, respectively. An example of the RT-QuIC output in the CSF of a sporadic CJD patient is given in Figure 1.

Finally, Sano and colleagues [44] reported that the RT-QuIC assay on the CSF of patients with genetic prion diseases has 78% sensitivity in GSS, 100% in FFI, 87% in E200K genetic CJD, and 100% in V203I genetic CJD, suggesting that the RT-QuIC assay for the detection of PrP^TSE^ in the CSF might become a valid method for improving the diagnosis of patients with a clinical suspicion of human prion disease. 

Besides CSF, the RT-QuIC assay revealed PrP^TSE^ in nasal lavages from hamsters infected with the transmissible mink encephalopathy (TME) hyper strain [40] and, by using immunoaffinity beads coupled with the conformational 15B3 anti- PrP^TSE^ antibody (enhanced QuIC), in plasma of scrapie-infected hamsters [39]. The assay showed 100% sensitivity and specificity and was able to detect a positive signal long before the appearance of clinical signs of scrapie.

Finally, the application of 15B3-conjugated beads to the QuIC protocol and the use of a hamster-sheep chimeric recPrP as substrate in the reaction increased the sensitivity (up to attogram levels) and the speed of detection (28 hrs) of variant CJD brain misfolded proteins spiked into human blood [39]. Despite this good achievement there is still no report on the use of the enhanced QuIC assay in human blood.

Overall, RT-QuIC methodology is a powerful platform for the detection and large-scale screening of misfolded PrP in both human and animals. Up to attogram levels of misfolded PrP can be detected and properly quantified within few hours by using high-throughput 96-well formats. The high levels of specificity obtained in a variety of tissues and species by using flexible recombinant substrates demonstrates the versatility of the novel method. It is of note that RT-QuIC PK-resistant products are reported to be noninfectious (quoted by [43]) and therefore likely more secure in large-scale screening diagnostic procedures. The two disadvantages of this assay are the relatively poor performance in amplifying PrP^TSE^ from variant CJD tissues [64] and the failure to reproduce the original PrP^TSE^ signature impeding the molecular diagnosis of sporadic CJD and the distinction between sporadic and variant CJDs.

## 4. Other Potential Assays for the Detection of Misfolded PrP in Blood

### 4.1. Immunocapillary Electrophoresis (ICE)

The assay, originally developed by Schmerr and colleagues [65] and based on a competitive immunoassay with PrP fluorescent peptides, was soon proven efficient for the detection of PrP^TSE^ in blood of scrapie-infected sheep and elks with CWD [66]. However, these results were not confirmed in other laboratories using blood samples from CJD-infected chimpanzees or sporadic, iatrogenic, genetic, and variant CJD patients [45, 67]. It is therefore unlikely that this assay will be of any use for the diagnosis of human prion diseases. 

### 4.2. Surface Fluorescence Intensity Distribution Analysis (Surface-FIDA)

This assay consists in the immobilization of single PrP aggregates on a capture antibody coated surface that are then visualized by the concomitant binding with two anti-PrP fluorescent antibodies and a double-laser beam scanning system (surface-FIDA). The method discriminates aggregated PrP forms from monomeric PrP without the use of the proteinase K (PK) digestion step and therefore recognizes both PK-resistant and PK-sensitive PrP^TSE^. Surface-FIDA enabled the counting of bovine and hamster PrP aggregates in brain homogenates and in bovine cerebrospinal fluid [47]. PrP aggregates were also blind-detected in blood of scrapie-infected sheep (*n* = 15) with high specificity and sensitivity [46], although it remains unsettle whether the detection of PrP aggregates correlates with infectivity. It is of note that spiking of blood plasma with PrP^TSE^ from brain was unsuccessful suggesting that the properties of PrP^TSE^ from brain are different from endogenous blood misfolded PrP [46].

### 4.3. Ligand-Based Immunoassay

Terry and colleagues [48] reported the detection of PrP^TSE^ in 55% of blood mononuclear cells (PBMC) obtained from scrapie-affected sheep (*n* = 80) and 71% of experimentally BSE-affected sheep by a modified polyanionic ligand assay of the IDEXX HerdCheck methodology [68]. The assay resulted positive also in a subset of scrapie-infected sheep several months before the onset of clinical signs suggesting that PrP^TSE^ can be detected in asymptomatic prion-infected hosts. However, the relatively low sensitivity observed in prion-infected sheep, the long timings of sample preparations, and the amount of blood volumes required for the purification of PBMC foretell that this assay would not be easily applicable to large-scale diagnostic scopes.

### 4.4. Solid-State Binding Matrix

The assay, based on the affinity that PrP^TSE^ has for stainless steel particles [69, 70], was adapted for the detection of misfolded PrP in blood of patients with various forms of CJDs [49]. The selective absorption of PrP^TSE^ on the metal matrix concentrates misfolded protein up to the point that the signal can be detected by an ELISA assay. Because of the selectivity of the metal matrix in binding only misfolded PrP, there is no need to pretreat samples with PK that likely removes a conspicuous fraction of PrP^TSE^ in blood. This method was initially tested on human blood spiked with vCJD brain homogenate where misfolded particles in up to the 10^−10^ brain dilution were detected. Subsequently, blood of variant and sporadic CJD patients was analysed on a blinded experiment including samples from patients with other neurological diseases and controls. Only samples that were reactive in two separate assays were scored as positive. About two-third of blood samples from variant CJD but none from sporadic CJD patients and neurological or nonneurological controls yield positive signals in both assays resulting in 100% specificity for variant CJD [49].

### 4.5. EP-vCJD Blood Screening Assay

In 2003, Paramithiotis and colleagues [71] reported the manufacture of an antibody directed against PrP epitopes that are exposed only upon protein misfolding and therefore specific to PrP^TSE^. This conformational anti-PrP^TSE^ antibody was then used for the epitope-protection (EP) vCJD-screening assay, which was later implemented by Amorfix. The high-throughput assay achieved 100% sensitivity and specificity on 1,000 blinded human plasma samples, which included samples that were spiked with variant CJD-infected and normal brains [50]. In 2009, the specificity of the method was ascertained on a large-scale screening initiated in France in over 20,000 human blood samples [51]. Results showed that on the first run 486 samples were positive (97.6% specificity), 20 of which were then confirmed positive on a second screening [51]. The repeat-reactive samples were finally considered negative on a third screening [51]. Subsequently, Amorfix tested three variant CJD blood samples provided by the National Institute for Biological Standards and Control (NIBSC, UK) that resulted negative [52]. The sensitivity of the test was therefore further improved for the detection of 1 : 5,000,000 dilution of variant CJD-infected brain spiked into blood [52]. However, despite this enhanced sensitivity, the test was still unable to detect prions in blood of variant CJD patients, and it was finally concluded that more research is required before the reevaluation of the assay [53].

### 4.6. Conformation-Dependent Immunoassay (CDI)

In 1998, Safar and colleagues [10] developed an ELISA-formatted, dissociation-enhanced time-resolved fluorescence detection system based on specific antibody binding to epitopes that are accessible in PrP^C^ but that are unmasked only in denatured PrP^TSE^. This method does not require PK treatment and is able to recognize both sensitive and resistant PK misfolded proteins and different PrP^TSE^ conformations. The assay, improved by incorporating a capture antibody, was able to discriminate PrP^TSE^ signature in different molecular forms of sporadic CJDs, iatrogenic CJDs and genetic TSEs [72] and detect up to a 10^−5^ dilution of PrP^TSE^ from variant CJD brain used for spiking human normal plasma [54, 73]. However, endogenous PrP^TSE^ was undetectable in white blood cells of sporadic patients by CDI [55], but we are not aware of its use in variant CJD blood.

### 4.7. Misfolded Protein Diagnostic Assay (MPD)

This technique is based on a pyrene-labeled palindromic sequence of prion peptides that converts to *β*-sheets in the presence of PrP^TSE^ [56, 74]. This process induces an excimeric signal from the conjugated pyrenes that propagates to other peptides with the final goal to amplify the PrP^TSE^ signal. MPD assay detects PrP^TSE^ in brain of 263K scrapie-infected hamsters during the preclinical and clinical stages of disease [74] and in small volumes of plasma from prion-infected mice and sheep with sensitivity up to 1 infectious dose per mL [56]. The same assay discriminated in blinded small-scale experiments control plasma from that of patients with sporadic CJD and squirrel monkeys with experimental CJD with 100% specificity and sensitivity [56]. 

### 4.8. Multimer Detection System (MDS)

This technique is a modified ELISA assay that recognizes only multimeric forms of PrP^TSE^ without using any pretreatment with proteinases, which might remove PrP^TSE^ forms likely present in body fluids [57]. This assay uses the same principle previously described by Pan and colleagues [75] and is based on the use of two monoclonal antibodies that share overlapping epitopes. Monomers (PrP^C^) are captured by an antibody attached to the surface of a plate and are not detected by the second antibody due to the absence of any exposed epitopes. On the other hand, multimers (PrP^TSE^) are easily recognized by the second antibody because they expose more copies of the same epitope. The assay was tested on plasma samples of nine scrapie-infected and nine control hamsters resulting in 100% specificity and sensitivity [57]. This simple assay, however, requires validation in other laboratories and more basic work for determining whether the multimeric forms detected by the MDS assay are related to infectivity.

## 5. Final Remarks

It is unquestionable that in the last 15 years there has been an outstanding progress in improving the detection of PrP^TSE^ for developing sensitive and specific diagnostic assays. These sophisticated and highly sensitive methods successfully detect up to attogram levels of PrP^TSE^ in body fluids of different species (Table 1). A major breakthrough is the development of the RT-QuIC technology for the detection of PrP^TSE^ in the CSF of patients with sporadic [43, 64] and genetic [44] TSEs that as soon as is validated by other groups will change the diagnostic criteria of human prion diseases. 

Endogenous PrP^TSE^ has been identified in blood of scrapie-infected hamster by PMCA [27, 28] and RT-QuIC [39] assays and of patients with variant CJD by the solid-state binding matrix assay [49]. Despite these successful observations, however, there are no published reports on the application of either PMCA or enhanced RT-QuIC on blood samples of patients with any form of prion diseases suggesting that both assays still need substantial improvement before their use in the diagnostic setting. Although the development of the RT-QuIC technology for the detection of PrP^TSE^ in blood samples is more recent than PMCA, an extra impediment of the RT-QuIC assay might come from the interference of blood molecules with the ThT reading. On the other hand, the solid-state binding matrix assay might be a valid alternative for the development of a blood test for variant CJD, but the relative low sensitivity (71%) and the finding that some control samples resulted positive in one of the two runs [49] make the use of this assay a remote ambition.

What remains elusive is the reproducible detection of endogenous PrP^TSE^ in blood despite the successful identification of minute amounts of spiked brain PrP^TSE^ into healthy blood. It becomes more and more evident that the properties of PrP^TSE^ in brain are different from those in blood and that some components of blood both inhibit and interfere with PrP^TSE^ detection causing false positive and negative results and compromising the reproducibility of the assay [46, 51, 76]. A clear example is given by the failure of the EP-vCJD assay that had excellent and reproducible performances on spiked blood but then completely failed to identify positive and negative human blood samples [50–53].

These findings pose the question on whether the criteria delineated by the National Institute for Biological Standards and Control (NIBSC, UK) [77] for prion diagnostic assay validation in terms of satisfactory sensitivity and specificity on spiked blood and for the request of variant CJD blood samples are still relevant for defining the best condition of success of potential prion test in blood.

We think that the principles for assay validation and accessibility to variant CJD blood samples should rather focus on reproducible and large scale blinded studies on blood taken from animal models of prion diseases, such as scrapie or BSE in sheep followed by a large scale screening of healthy blood donors to ascertain a sufficient level of specificity. 

Finally, our impression is that the research on prion detection in blood does not really need further sensitive assays but rather requires further work aiming to the identification of interfering blood components and understanding prion metabolism in blood.

## Figures and Tables

**Table 1:** Detection of misfolded PrP in body fluids.

Assay^1^	Species	Body fluid	Assay sensitivity (brain dilution)^2^	Inoculation route	Time (dpi)	Starting volume (mL)	Number of tested samples (treated/controls)	Sensitivity (%)	Specificity (%)	Reference
					14	1	5/5	0	100	
					20	1	6/4	50	100	
					40	1	10/5	60	100	
			10^−12^ (fg)	ip	60	1	5/4	40	100	[28]
		*Blood (bc) *			70	1	5/5	20	100	
					80	1	5/5	0	100	
	*Hamster *				90 (c.s.)	1	10/10	80	100	
			10^−12^ (fg)	ic	68 (c.s.)	1	18/12	89	100	[27]
			10^−12^ (fg)	ic	60	1.5	4/3	100	100	[29]
					68 (c.s.)	1.5	3/3	100	100	
		*Blood (plasma) *	10^−12^ (fg)	ic	60	1.5	4/5	50	100	[29]
					68 (c.s.)	1.5	4/5	67	100	
		*Urine *	10^−12^ (fg)	ic	68 (c.s.)	0.02	7/4	86	100	[29]
				oi	155 (c.s.)	0.02	7/4	67	100	
			(—)	ip	90 (c.s.)	(—)	5/5	80	100	[30]
PMCA		*CSF *	(ag)	ic	68 (c.s.)	0.002	6/14	100	100	[32]
					30	10^4^	1/2	0	100	
					60	10^+4^ (cells)	1/2	0	100	
		*Blood (wbc) *	10^−8^	oi	90	10^+4^ (cells)	1/2	100	100	[33]
					130	10^+4^ (cells)	1/2	100	100	
					190 (c.s.)	10^+4^ (cells)	1/2	100	100	
	*Sheep *		10^−9^	ni	c.s.	10^+7^ (cells)	10/8	100	100^3^	[20]
		*Blood (plasma) *	10^−8^	ni/oi^4^	b.c.s.	0.5	6/7	100	100	[34]
					c.s.	0.5	9/7	100	100	
		*Urine *	10^−8^	ni/oi^4^	c.s.	0.5	4/4	100	100	[35]
		*Saliva *	(fg)	ni	360	(—)	12/20	100	88	[36]
	*Cattle *	*Saliva *	10^−11^ (fg)	oi	c.s.	0.005	3/1	33	100	[37]
		*CSF *	10^−11^ (fg)	oi	c.s.	0.005	3/1	33	100	
		*Blood (plasma) *	10^−8^	oi	b.c.s.	0.5	2/1	100	100	[34]
	*Cervids *				c.s.	0.5	3/1	100	100	
		*Urine *	10^−8^	oi	c.s.	0.5	5/2	100	100	[35]
		*CSF *	10^−5^	ni	c.s.	0.025	4/31	75	100	[38]

					10	0.5	1/11	100	100	
		*Blood (plasma) *	4 × 10^−14^ (ag)^5^	ic	30	0.5	3/11	100	100	[39]
	*Hamster *				80 (c.s.)	0.5	9/11	100	100	
		*CSF *	10^−9^	ic	c.s.	0.002	2/2	100	100	[40]
		*Nasal lavage *	10^−9^	ic	c.s.	0.004	8/2	100	100	[40]
	*Sheep *	*CSF *	(fg)	ni	c.s.	0.005	1/1	100	100	[41]
QUIC		*Spiked vCJD blood (plasma) *	4 × 10^−14^ (ag)^5^	ni	c.s.	0.5	4/4	100	100	[39]
		*sCJD CSF *	10^−9^ (fg)^6^	ni	c.s.	0.005	16/14	87.5	100	[42]
			5 × 10^−6^ (0.1 pg)	ni	c.s.	0.015	67/51	87	100	[43]
	*Human *	*gCJD CSF (E200K) *	10^−9^ (fg)^6^				22/1	87	100	
		*gCJD CSF (V203I) *	10^−9^ (fg)^6^	ni	c.s.	0.005	2/1	100	100	[44]
		*GSS CSF *	10^−9^ (fg)^6^				20/1	78	100	
		*FFI CSF *	10^−9^ (fg)^6^				12/1	100	100	

ICE	*Human *	*CJD Blood (bc) *	(—)	ni	c.s.	10	9/6	55	100	[45]

sFIDA	*Sheep *	*Blood (plasma) *	(—)	ni	c.s.	(—)	10/5	100	100	[46]
	*Cattle *	*CSF *	(—)	oi	c.s.	0.02	6/6	50	100	[47]

Ligand-based IA		*Blood (wbc) *	(—)	ni	b.c.s.	5	23/129	56	100	
	*Sheep *	*Blood (wbc) *	(—)	ni	c.s.	5	80/129	55	100	[48]
		*Blood (wbc) *	(—)	oi	c.s.	5	7/129	71	100	

Metal matrix	*Human *	*vCJD blood *	10^−10(5)^	ni	c.s.	(—)	21/190	70	100	[49]
		*sCJD blood *					27/190	0	100	

EP-vCJD	*Human *	*Spiked vCJD blood *	5 × 10^−7(5)^	ni	c.s.	(—)	nd/1000	100	100	[50]
		*vCJD blood *		ni	c.s.	(—)	3/20000	0	98	[51–53]

CDI	*Human *	*vCJD blood *	10^−5(5)^	ni	c.s.	(—)	7/4	100	100	[54]
		*sCJD blood (wbc) *	0.5 pg	ni	c.s.	10^+6^ cells	24/27	0	100	[55]

MPD	*Sheep *	*Blood (serum) *		ni	c.s.	0.2	13/1	100	100	[54]
	*Primates *	*Blood (plasma) *	1 ID/mL^7^	ni	c.s.	0.2	8/4	100	100	[56]
	*Human *	*sCJD Blood (plasma) *		ni	c.s.	0.2	5/5	100	100	

MDS	*Hamster *	*Blood (plasma) *	10^−8^	(—)	c.s.	0.078	9/9	100	100	[57]

^1^The assay specification does not include protocol variations.

^2^Assay sensitivity is indicated as the end point dilution of scrapie brain homogenates. In brackets, estimates of the PrP^TSE^ amount provided by the authors.

^3^Spontaneous formation of misfolded PrP was reported in some control samples after increased cycles of sonication.

^4^Authors used samples from both naturally and experimentally infected sheep.

^5^Dilution of variant CJD brain into healthy human blood.

^6^Dilution of sporadic CJD into artificial CSF.

^7^Measured on previously titered terminal plasma pool from mice experimentally infected with the Fukuoka-1 strain of GSS.

dpi: days postinoculation; fg: femtograms; ag: attograms; bc: buffy coat; wbc: white blood cells; c.s.: clinical symptoms; b.c.s.: before clinical symptoms; ic: intracerebral; ip: intraperitoneal; oi: oral inoculation; ni: natural infection; CSF: cerebral spinal fluid; CJD: Creutzfeldt-Jakob disease; GSS: Gerstmann-Sträussler-Scheinker syndrome; FFI: fatal familial insomnia.
